# Molecular characterization of multidrug-resistant Gram-negative pathogens in three tertiary hospitals in Cairo, Egypt

**DOI:** 10.1007/s10096-020-03812-z

**Published:** 2020-01-17

**Authors:** Amani A. El-Kholy, Samia A. Girgis, Mervat A. F. Shetta, Dalia H. Abdel-Hamid, Arwa R. Elmanakhly

**Affiliations:** 1 Department of Clinical Pathology, Faculty of Medicine, Cairo University, Giza, Egypt; 2 Department of Clinical Pathology, Faculty of Medicine, Ain Shams University, Cairo, Egypt; 3 Department of Clinical Pathology, Ain Shams Specialized Hospital, Cairo, Egypt; 4 Department of Microbiology and Infection Control, Dar-Al-Fouad Hospital, Cairo, Egypt

## Abstract

High rates of antimicrobial resistance (AMR) among Gram-negative pathogens (GNP) have been reported in Egypt. Antimicrobial surveillance and identifying the genetic basis of AMR provide important information to optimize patient care. In this study, we aimed to identify the beta-lactam resistance phenotypes and genotypes of multidrug-resistant (MDR) non-repetitive GNP from 3 tertiary hospitals in Egypt. WZe studied 495 non-repetitive MDR Gram-negative isolates from patients with complicated intra-abdominal infections (cIAI), complicated urinary tract infection (cUTI), and lower respiratory tract infection (LRTI), collected as part of the “Study for Monitoring Antimicrobial Resistance Trends” (SMART) conducted in 3 tertiary hospitals in Cairo, Egypt, from 2015 to 2016. Identification and susceptibility testing of GNP to antimicrobials were tested in each hospital laboratory and confirmed in a reference laboratory (International Health Management Associates (IHMA), Inc., Schaumburg, IL, USA). Molecular identification of extended-spectrum beta-lactamases (ESΒLs), AmpC, and carbapenem resistance genes was conducted in IHMA. Among the 495 MDR isolates, *Klebsiella pneumoniae* (*K*. *pneumoniae*) and *Escherichia coli* (*E*. *coli*) were the most common (52.7% and 44.2%). *K*. *pneumoniae* was most susceptible to colistin, amikacin, ertapenem, and imipenem (92.7%, 72.7%, 69.3%, and 64%, respectively). *E*. *coli* was most susceptible to colistin (100%), amikacin (94.1%), imipenem (90.4%), and ertapenem (83.6%). ESBL was detected in 96.2% and ESBL genotypes included *bla*_CTX-M-15_ (70.1%), *bla*_TEM-OSBL_ (48.5%), *bla*_SHV-OSBL_ (27.9%), and *bla*_CTX-M-14_ (10.7%). AmpC resistance genes were identified in 9.7% of the isolates, dominated by *bla*_CMY-2_ (5.7%)**.** Carbapenem resistance genes were detected in 45.3% of the isolates. In *K*. *pneumoniae*, *bla*_OXA-48_ dominated (40.6%), followed by *bla*_NDM-1_ (23.7%) and *bla*_OXA-232_ (4.5%). In *E*. *coli*, the most frequent genes were *bla*_NDM-5_ (9.6%), *bla*_OXA-181_ (5.5%), *bla*_OXA-244_ (3.7%), and *bla*_*NDM-1*_ (3.7%). *bla*_*KPC-2*_ was identified in 0.4% of isolates. Notably, 32.3% of isolates carried more than one resistance gene. Our findings emphasize the continued need for molecular surveillance of MDR pathogens, implementation of strict infection control measures, and antimicrobial stewardship policies in our hospitals.

## Introduction

Antimicrobial resistance (AMR) among Gram-negative pathogens (GNP) increased worldwide. A high rate of AMR has been reported in Egypt since more than 20 years, among GNP causing nosocomial infections and outbreaks [1–3]. AMR rates have increased especially among nosocomial GNP, probably due to widespread abuse of antimicrobials including carbapenems in Egyptian hospitals and poor compliance with infection control practices [4, 5]. In small-scale studies, *bla*_*OXA*_, *bla*_*NDM*_, *bla*_*VIM*_, *bla*_*IMP*_, and *bla*_*KPC*_ carbapenemase genes were detected in Egypt [6–8]. As the genetic basis of beta-lactam resistance was not yet studied at a large scale in Egypt, we aimed to molecularly characterize multidrug-resistant (MDR) GNP.

## Methods

### Study sites and strains

This study was conducted in 3 major tertiary care Egyptian hospitals participating in the “Study for Monitoring Antimicrobial Resistance Trends” (SMART) from 2015 to 2016. The hospitals were Ain Shams University Hospital, Ain Shams Specialized University Hospital, and Dar Al-Fouad Hospital. Isolates were collected according to SMART protocol as previously reported [9–11]. Briefly, the participating hospitals collected 1070 non-repetitive consecutive GNP isolates from lower respiratory tract specimens, urine and pus or abdominal fluid of hospitalized patients with lower respiratory tract infections (LRTI), complicated urinary tract infections (cUTI), and complicated intra-abdominal infections (cIAI) during the study period. Of these, we studied 495 isolates that showed phenotypic resistance to third-generation cephalosporins or carbapenems. The identification of GNP, susceptibility testing, and detection of resistance phenotypes were conducted in hospital laboratories according to the methods of the Clinical and Laboratory Standards Institute [12] and confirmed in a reference laboratory (International Health Management Associates (IHMA), Inc., Schaumburg, IL, USA), where susceptibility and extended-spectrum β-lactamase (ESBL) phenotype were determined using the CLSI broth microdilution method with custom dehydrated panels manufactured by Trek Diagnostic Systems (Thermo Scientific, Independence, OH) in 2015–2016. MIC interpretive criteria followed the 2017 M100-S27 guidelines of the CLSI [13]. EUCAST breakpoints were used only for colistin against *Enterobacteriaceae*, because no CLSI breakpoints exist [14].The susceptibility of all Gram-negative isolates combined was calculated using breakpoints appropriate for each species.

### Genotypic identification of antimicrobial resistance genes

The molecular characterization of ESBL and carbapenemases was done using the Check-Points MDR CT103 (Check-Points Health B.V., Wageningen, The Netherlands) microarray kit, which detects most carbapenemase, ESBL, and AmpC genes: ESΒLs (class A)—*bla*_TEM_, *bla*_*SHV*_, *bla*_*CTX-M*_, *bla*_*VEB*_, *bla*_*PER*_, and *bla*_*GES*_; *bla*_*AmpC*_ β-lactamase genes (class C)—*bla*_*ACC*_, *bla*_*ACT*_, *bla*_*CMY*_, *bla*_*DHA*_, *bla*_*FOX*_, *bla*_*MIR*_, and *bla*_*MOX*_; and carbapenemases (class A)—*bla*_*KPC*_ and *bla*_*GES*_; (class B)—*bla*_*NDM*_, *bla*_*IMP*_, *bla*_VIM_, *bla*_*GIM*_, and *bla*_*SPM*_; and (class D)—*bla*_*OXA-48-like*_. Then the genes encoding ESΒL, carbapenemases, and AmpC were sequenced in their entirety in IHMA [11].

## Results

### Strains and phenotypic antibiotic susceptibility

The 495 MDR isolates were derived from cIAI (181, 36%), LRTI (128, 26%), and cUTI (186, 38%). Overall *Klebsiella pneumoniae* and *Escherichia coli* were the most common (52.7% and 44.2%) and were also the predominant organisms in cIAI (52.2% and 45.9%, respectively), cUTI (43% and 54.8%, respectively), and LRTI (71.1% and 26.6%, respectively) (Table 1). *K*. *pneumoniae* was most susceptible to colistin, amikacin, ertapenem, and imipenem (92.7%, 72.7%, 69.3%, and 64%, respectively). *E*. *coli* remained most susceptible to colistin (100%), amikacin (94.1%), imipenem (90.4%), and ertapenem (83.6%).

**Table 1:** Distribution of microorganism among clinical isolates

Organisms	IAI		UTI		LRT	
	No.	%	No.	%	No.	%
*Escherichia coli* (219)	83	45.9	102	54.8	34	26.6
*Klebsiella pneumoniae* (266)	95	52.5	80	43.0	91	71.1
Others (10)	*3*	*1.7*	*4*	*2.2*	*3*	*2.3*
Total (495)	181		186		128	

### Identification of *bla*_ESBL_ genes of the TEM, SHV, and CTX-M types

ESBL production was detected in 96.2% of the MDR isolates (Table 2). ESBL genotypes included *bla*_CTX-M-15_ (70.1%), *bla*_TEM-OSBL_ (48.5%), *bla*_SHV-OSBL_ (27.9%), and *bla*_CTX-M-14_ (10.7%). The predominant ESBL gene in both *E*. *coli* and *K*. *pneumoniae* was *bla*_*CTX-M-15*_ (Table 3).

**Table 2:** Percentage of ESΒL, AmpC β-lactamases, and carbapenemase genes among 495 MDR isolates

Resistance genes	Number	Percentage
ESΒL	474	96.2
AmpC	48	9.7
Carbapenemase	224	45.3

**Table 3:** ESΒL predominant genotypes among *E*. *coli* and *K*. *pneumoniae*

	SHV no. (%)		TEM no. (%)		CTX-M-1 no. (%)		CTX-9 no. (%)	
	SHV-OSBL	SHV-12	TEM-OSBL	TEM-ESΒL	CTX-M-55	CTX-M-15	CTX-M-27	CTX-M-14
*E*. *coli* (219)	1	7	104	2	2	150	2	1
	(0.5)	(3.2)	(47.5)	(4.2)	(4.2)	(68.5)	(0.9)	(0.5)
*K*. *pneumoniae* (266)	136	34	129	1	0	190	12	52
	(51.1)	(12.8)	(48.5)	(0.4)	0.0	(71.4)	(4.5)	(19.5)
Others (10)	1	1	7	0	0	7	0	9
	(10)	(10)	(70)	(0)	(0)	(70)	(0)	(90)
Total (495)	138	42	240	3	2	347	14	53
	(27.9)	(8.5)	(48.5)	(0.6)	0.4	(70.1)	(2.8)	(10.7)

### Identification of carbapenemase genes

Carbapenem resistance genes were detected in 45.3% of the MDR isolates. In *K*. *pneumoniae*, *bla*_*OXA-48*_ dominated (40.6%), followed by *bla*_*NDM-1*_ (23.7%) and *bla*_*OXA-232*_ (4.5%). In *E*. *coli*, the most frequent genes were *bla*_*NDM-5*_ (9.6%), *bla*_*OXA-181*_ (5.5%), *bla*_*OXA-244*_ (3.7%), and *bla*_*NDM-1*_ (3.7%). *blaKPC-2* and *bla*_*VIM-2*_ were less frequently identified (Table 4).

**Table 4:** Carbapenemase genotypes among *E*. *coli* and *K*. *pneumoniae*

Carbapenemase genes	KPC no. (%)	OXA no. (%)			MBL no. (%)				VIM no. (%)
	KPC-2	OXA-48	OXA-244	OXA-232	OXA-181	NDM-1	NDM-4	NDM-5	
*E*. *coli* (219)	0	5	8	0	12	8	1	21	1
	(0)	(2.3)	(3.7)	(0)	(5.5)	(3.7)	(0.5)	(9.6)	(0.5)
*K*. *pneumoniae* (266)	2	108	0	12	3	63	1	8	3
	(0.8)	(40.6)	(0)	(4.5)	(1.1)	(23.7)	(0.4)	(3.0)	(1.1)
Others (10)	0	2	0	0	0	3	0	1	1
	(0)	(20)	(0)	(0)	(0)	(30)	(0)	(10)	(10)
Total (495)	2	115	8	12	15	74	2	30	5
	(0.4)	(23.2)	(1.6)	(2.4)	(3.0)	(14.9)	(0.4)	(6.1)	(1.0)

### Identification of AmpC β-lactamases resistance genes

AmpC resistance genes were identified in 9.7% of the isolates; *bla*_CMY-2_ was the most predominant one (Table 5). In 153 isolates (32.3%), coexistence of 2 or more resistance genes was detected (Table 4). The commonest combination of 2 genes was *bla*_*CTX-M-15*_ with *bla*_*NDM-5*_ (2.6%); the commonest combination of 3 genes was *bla*_*SHV-OSBL*_, *bla*_*CTX-M-15*_, and *bla*_*NDM-1*_ (9.2%); the commonest combination of 4 genes was *bla*_*SHV-OSBL*_, *bla*_*TEM-OSBL*_, *bla*_*CTX-M-15*_, and *bla*_*NDM-1*_ (12.4%); and the commonest combination of more than 4 genes was *bla*_*SHV-OSBL*_, *bla*_*TEM-OSBL*_, *bla*_*CTX-M-15*_, *bla*_*CTX-M-14*_, *bla*_*NDM-1*_, and *bla*_*OXA-48*_ (5.9%) (Table 6). Tables 7 and 8 show K. pneumoniae and E.coli susceptibility (%) against MDR- GNP per type of infection.

**Table 5:** Total AmpC β-lactamases genotype among *Enterobacteriaceae* isolates

CMY II no. (%)						DHA-1	ACT-TYPE
CMY-2	CMY	CMY-TYPE	CMY-4	CMY-42	CMY-59		
28	2	3	1	3	2	6	3
(5.7)	(0.4)	(0.6)	(0.2)	(0.6)	(0.4)	(1.2)	(0.6)

**Table 6:** Common combinations of resistant genes among tested isolates

Types of combination	No. of isolates	Percentage
2 combination	*15*	*9.8*
*bla*_*CTX*_*-*_*M-15*_*; bla*_*NDM-5*_	4	2.6
3 combinations	*44*	*28.8*
*bla*_*SHV-OSBL*_*; bla*_*CTX-M-15*_*; bla*_*NDM-1*_	14	9.2
*bla*_*SHV-OSBL*_*; bla*_*CTX-M-14*_*; bla*_*OXA-48*_	8	5.2
*bla*_*SHV-OSBL*_*; bla*_*CTX-M-15*_*; bla*_*OXA-48*_	7	4.6
4 combinations	*70*	*45.8*
*bla*_*SHV-OSBL*_*; bla*_*TEM-OSBL*_*; bla*_*CTX-M-15*_*; bla*_*NDM-1*_	19	12.4
*bla*_*SHV-OSBL*_*; bla*_*CTX-M-15*_*; bla*_*CTX-M-14*_*; bla*_*OXA-48*_	11	7.2
*bla*_*SHV-12*_*; bla*_*CTX-M-15*_*; bla*_*CTX-M-27*_*; bla*_*OXA-48*_	8	5.2
*bla*_*SHV-OSBL*_*; bla*_*TEM-OSBL*_*; bl*_*CTX-M-14*_*; bla*_*OXA-48*_	6	3.9
*bla*_*TEM-OSBL*_*; bla*_*CTX-M-15*_*; bla*_*CMY-2*_*; bla*_*NDM-5*_	6	3.9
*bla*_*SHV-OSBL*_*; bla*_*CTX-M*_*-15; bla*_*NDM-1*_*; bla*_*OXA-48*_	5	3.3
*bla*_*SHV-OSBL*_*; bla*_*CTX-M-14*_*; bla*_*NDM-1*_*; bla*_*OXA-48*_	4	2.6
> 4 combinations	*22*	*14.4*
*bla*_*SHV-OSBL*_*; bla*_*TEM-OSBL*_*; bla*_*CTX-M-15*_*; bla*_*CTX-M-14*_*; bla*_*NDM-1*_*; bla*_*OXA-48*_	9	5.9
*bla*_*SHV-OSBL*_*; bla*_*CTX-M-15*_*; bla*_*CTX-M-14*_*; bla*_*NDM-1*_*; bla*_*OXA-48*_	5	3.2

**Table 7:** *K*. *pneumoniae* susceptibility (%) against MDR-GNP per type of infection

Body site	AK	AMC	FEP	CTX	FOX	CTZ	CRO	CST	ETP	IMP	LEV	TZP
IAI	76.5	13.4	8.1	0	19.4	3.1	0	93.7	74.5	58.2	25.5	20.4
LRTI	57.5	0	2.3	1.2	17.2	2.3	1.2	89.7	51.7	75.8	20.7	16.1
Urine	84.2	1.9	5.7	1.4	14.3	5.7	1.4	97.1	81.4	60	18.6	18.6
Total	*72.7*	5.6	5.6	0.7	16.7	3.4	0.8	*92.7*	*69.3*	*64.0*	22	18.6

*AK* amikacin, *AMC* ampicillin/sulbactam, *FEP* cefipeme, *CTX* ceftriaxone, *FOX* cefoxtine, *CTZ* ceftazidieme, *CRO* cefotaxime, *CST* colistin, *ETP* ertapenem, *IMP* imipenem, *LEV* levofloxacin, *TZP* piperacillin/tazobactam

**Table 8:** *E*. *coli* susceptibility (%) against MDR-GNP per type of infection

Body site	AK	AMC	FEP	CTX	FOX	CTZ	CRO	CIP	CST	ETP	IMP	LEV	TZP
IAI	91.6	6.8	0	0	12.1	10.8	0	24.1	100	84.3	86.7	25.3	43.4
LRTI	91.2	18.2	0	0	17.7	11.8	0	17.7	100	79.4	91.2	17.7	41.2
Urine	96.8	14.3	1.1	0	16	6.4	0	11.7	100	84.1	92.6	11.7	56.4
Total	*94.1*	7.3	0.4	0	14.2	9.1	0	17.8	*100*	83.6	*90.4*	18.3	48.9

*AK* amikacin, *AMC* ampicillin/sulbactam, *FEP* cefipeme, *CTX* ceftriaxone, *FOX* cefoxtine, *CTZ* ceftazidieme, *CRO* cefotaxime, *CST* colistin, *ETP* ertapenem, *IMP* imipenem, *LEV* levofloxacin, *TZP* piperacillin/tazobactam

## Discussion

Surveillance for AMR is essential to monitor trends, identify emerging resistance mechanisms, and support the antimicrobial stewardship programs. To our knowledge, this is the most extensive molecular study of AMR in Egypt including 495 MDR Gram-negative isolates. In all hospitals, more than half of infections were present on admission (community acquired or transferred from other hospitals; data not shown). We identified 43 resistance phenotypes distributed among isolates from the 3 hospitals. Typing of nosocomial GNP in each hospital based on the phenotypic resistance patterns showed no clonal spread except for few isolates in each hospital (data not shown).

*K*. *pneumoniae* was a common pathogen in all 3 types of infection, and most isolates were MDR. Due to its large accessory genome including plasmids and chromosomal loci, *K*. *pneumoniae* isolates may act as opportunistic pathogens. Such strains infect critically ill and immunocompromised patients mostly, whereas other strains of *K*. *pneumoniae* (hyper-virulent) may even infect healthy people in community settings. Many of the virulent strains encode carbapenemases [15]. *E*. *coli* was a frequently identified pathogen from cIAI and cUTI, which is consistent with previous reports, and was also a frequent pathogen in LRTI. *E*. *coli* pneumonia is uncommon and may result from micro-aspiration of colonized upper airway secretions in severely ill patients; hence, it is a well-known cause of nosocomial pneumonia [16]. However, *E*. *coli* pneumonia may also be community-acquired in patients who have underlying diseases such as diabetes mellitus, alcoholism, chronic obstructive pulmonary disease, and *E*. *coli* UTI [17]. Our results confirm the results of previous studies in Egypt [3, 18] that showed high rates of ESΒL and carbapenem resistance among ICU pathogens. For example, in the study of Talaat et al. [18], ESBL and carbapenem resistance were identified in 54% and 13.8% of *E*. *coli* isolates compared with 42.5% and 48.1% in *K. pneumoniae* isolates, respectively.

Among the many types of ESΒLs reported, *bla*_*CTX-M-15*_ and *bla*_*CTX-M-14*_ are the most commonly identified worldwide as the genes encoding CTX-M enzymes (*bla*_CTX-M_) can be horizontally mobilized by various genetic elements [19]. This reflects the situation in Egypt as well. AmpC genes were less frequently identified (9.7%), usually in combination with other resistance genes. CMY-2-like enzymes were the most predominant. Other detected genes were *bla*_*CMY-42*_, *bla*_*DHA*_, and *bla*_*ACT-like*_. This is in keeping with recent reports on acquisition of plasmid-mediated cephalosporinase producing *Enterobacteriaceae* after a travel to the tropics and North Africa including Egypt [20, 21]. The high rate of ESΒL in Egypt is essentially due to inappropriate use of antimicrobials in human and animal health care. Of the patients treated in public outpatient health care facilities, 49.8% received antibiotics, and antibiotics are still sold over the counter without prescription [22]. Moreover, there is extensive use of antimicrobials for prevention and treatment of infections in veterinary care, and ESΒL and AmpC resistance mechanisms were detected in veterinary *E*. *coli* isolates in Egypt [23]. This is an urgent public health problem especially with a growing body of evidence supporting foodborne transmission of resistance, especially from poultry, as poultry meat exhibits the highest levels of contamination by MDR bacteria [24, 25].

The most important mechanism of carbapenem resistance is the production of carbapenemases; therefore, all isolates were investigated to identify the carbapenemase genes. We identified them in 45.4% of tested isolates; *bla*_*OXA*_ followed by *bla*_*NDM*_ genes dominated, while only 2 isolates of *K*. *pneumoniae* harbored *bla*_*KPC-*2_ genes. These results confirm previous small-scale reports that the *bla*_*NDM*_ and *bla*_*OXA*_ genes are the predominant in Egypt and Middle East [26].

The present study detected massive coexistence of different resistance genes among tested isolates. This coexistence could have contributed to the observed elevated variability in resistance phenotypes and genotypes among GNP in Egypt [15].

In conclusion, our study detected alarming rates of resistance and identified many resistance mechanisms in clinical GNP from Egyptian tertiary care hospitals. These high resistance rates highlight the importance of continuous monitoring of the resistance trends, adherence to infection control policies, and underscore urgently implementing a national antimicrobial stewardship plan in Egypt.
